# Efficiency Enhancement Mechanism for Poly(3, 4-ethylenedioxythiophene):Poly(styrenesulfonate)/Silicon Nanowires Hybrid Solar Cells Using Alkali Treatment 

**DOI:** 10.1186/s11671-016-1450-5

**Published:** 2016-05-25

**Authors:** Yurong Jiang, Xiu Gong, Ruiping Qin, Hairui Liu, Congxin Xia, Heng Ma

**Affiliations:** College of Physics & Materials Science, Henan Province Key Laboratory of Photovoltaic Materials, Henan Normal University, Xinxiang, 453007 China

**Keywords:** Silicon nanowire array, Alkali treatment, Core-shell, Hybrid solar cells

## Abstract

The efficiency enhancement mechanism of the alkali-treated Si nanowire (SiNW) solar cells is discussed and analyzed in detail, which is important to control the useful photovoltaic process. All the results demonstrate that the photovoltaic performance enhancement of alkali-treated SiNW device steps from the formation of the good core-shell heterojunction, which consequently enhances the junction area, promotes fast separating and transporting of electron and hole pairs, and reduces the carrier surface combination. It also indicates that alkali treatment for SiNWs is a promising processing as an economical method for the formation of good core-shell SiNW/polymer heterojunction.

## Background

Organic conjugated polymer/Si hybrid solar cells have received intensive attention due to their promising features, such as low-cost processing, light weight, low-temperature process, and large junction area [1–3]. Among the conjugated molecules, poly(3,4-ethylene dioxythiophene):poly-(styrenesulfonate) (PEDOT:PSS) is the commonly used conjugated polymer for the conjugated molecules/Si hybrid device [4–9]. PEDOT:PSS plays a major role as a hole transporting path as well as for the formation of heterojunction with Si [10].

Si nanowires (SiNWs) in the hybrid solar cells have been used for enhancing the light harvest in devices and could overcome high reflection issue effectively [11, 12]. Moreover, the large PEDOT:PSS/Si junction area enhances charge collection by shortening the paths traveled by minority carriers. To date, the PCEs of hybrid solar cells composed of SiNWs and PEDOT:PSS have been developed recently up to 14 % [13, 14].

However, the difficulty lies in the preparation of highly efficient core/shell nanowire photovoltaic junction in a simple way. Since the gaps between SiNWs are normally too small to be filled with the conductive polymer, If PEDOT:PSS do not infiltrate into gaps, then the photoexcited carriers are easily trapped by high-density surface defects owing to the large non-passivated surface area of nanostructures before being collected by electrodes [1]. PEDOT:PSS and the Si nanostructures are needed to allow polymers to infiltrate and form thin films on the SiNW surface. To this regard, the tapered Si nanostructures allowed for conformal polymer surface coverage via spin coating have been exploited [15, 16].

To obtain the tapered SiNWs in a simple way, the alkali treatment process was proposed [17], which easily tapered and separated the SiNWs from the bundled nanowires in a simple, cost-effective way, and the tapered SiNWs also demonstrated the improved photovoltaic characteristics. In our previous work, we found the phenomena that the alkali treatment process also could tapper the SiNWs and improve efficiency of the SiNW/PEDOT:PSS hybrid solar cells [18], then we attributed photovoltaic improvement to the good filling of PEDOT:PSS. However, our previous work just found the phenomena and deduced the conclusion from the morphology of scanning electronic microscopy (SEM) images; it still demands further in-depth investigations to provide more evidences and understand how alkali treatment processing could improve the photovoltaic performance in the organic/inorganic hybrid solar cells, which plays an important role in controlling the useful photovoltaic process.

In this work, we find the photovoltaic improvement of the alkali-treated SiNW/PEDOT:PSS device stems from the formation of good core-shell PEDOT:PSS/SiNW heterojunctions; the alkali-treated process provides superior coverage of PEDOT:PSS down to the bottom of the NWs, then enlarges PEDOT:PSS/Si junction area and enhances charge collection by shortening the paths traveled by the electron and hole pairs (EHPs). Our major investigation is to understand how the alkali treatment processing can dramatically improve the photovoltaic performance. The facile processing of alkali treatment is comparable to the chemical polish etching (CPE) and dry etching processing, which could promote the filling of PEDOT:PSS and formation of good core-shell heterojunction [15, 19].

## Methods

### Fabrication of SiNWs Combining Metal-assisted Etching and Alkali Treatment

Nanostructures were fabricated on n-type, polished (100) oriented Si wafers with 500-μm thickness and 1 to 3-Ω/cm resistivity. Firstly, silicon wafers were ultrasonically vibrated in acetone and ethanol at room temperature for 10 min to remove organic contaminations, respectively. The cleaned silicon wafers were immediately immersed in a mixed solution of deionized water, HF (4.6 mol/L) and AgNO_3_ (0.02 mol/L) for 3 min to grow nanostructures. The samples after etching were found to be wrapped with thick dendritic Ag structure [20, 21]. Silver nanoparticles were easily removed using nitric acid (68 wt%) dipping for 20 min after completion of the electroless etching. The samples after Ag removal were then rinsed with de-ionized water followed by dipping in 10 wt% HF solutions to remove the native oxide, rinsed with de-ionized water again, and dipped in solution of 2.5 M NaOH at 25 °C for 30 s.

### Hybrid Solar Cell Fabricating

Prior to device fabrication, the produced textured samples went through a rigorous cleaning procedure. At first, the samples were cleaned by immersing them in a solution consisting of H_2_O_2_ (30 %), H_2_SO_4_ (68 %), and DI water in the volume ratio of 1:1:5 at 120 °C for 10 min to remove any other organic residues. The samples were transferred to a DI water bath for 10 min. Again, the samples were immersed in a solution comprised of H_2_O_2_ (30 %), HCl (37 %), and DI water in the volume ratio of 1:1:5 at 80 °C for 10 min to remove any metallic contamination. The samples were then transferred to a DI water bath for 10 min. Finally, the samples were cleaned in a diluted HF (2 %) solution for 60 s to remove the native oxide and subsequently treated in UV-ozone cleaner for 15 min to improve the hydrophilic nature; this processing obviously improves the photovoltaic performance from 6.8 % in our previous work [15] to 9.1 %, the reason would be further investigated in the future. Immediately, the highly conductive PEDOT:PSS (Clevios PH500) mixed with 5 wt% dimethyl sulphoxide and 1 wt% Triton X-100 (surfactant) solution was spin-cast at 500 rpm for 15 s and 6000 rpm for 45 s to form a core-shell radial junction. The samples were then annealed on a hot plate at 140 °C for 10 min to remove the solvent. Finally, a 10-nm Cu film was deposited through a shadow mask (5 mm × 5 mm) as the top anode, a 200-nm-thick Al film was deposited on the backside for cathode contact.

### Characterization

Nanoscale surface and cross-sectional morphology of the SiNWs were characterized using a SEM (SUPRA™ 40). The morphology of single nanowire was characterized using the transmission electron microscope (TEM). The reflectivity of the substrates was measured by a UV-3600 spectrophotometer with an integrating sphere in the wavelength range of 300–1200 nm. The internal quantum efficiency (IQE) measurements were carried out by using a calibrated Newport 818-UV sensor and a monochromator (Acton Spectra Pro 2300i) with a lock-in amplifier (SR-830) and a SR540 optical chopper. The photovoltaic current density-voltage (*J*-*V*) characteristics of the solar cells were measured at air mass (AM) 1.5 G illumination.

## Results and Discussion

**Fig. 1:**
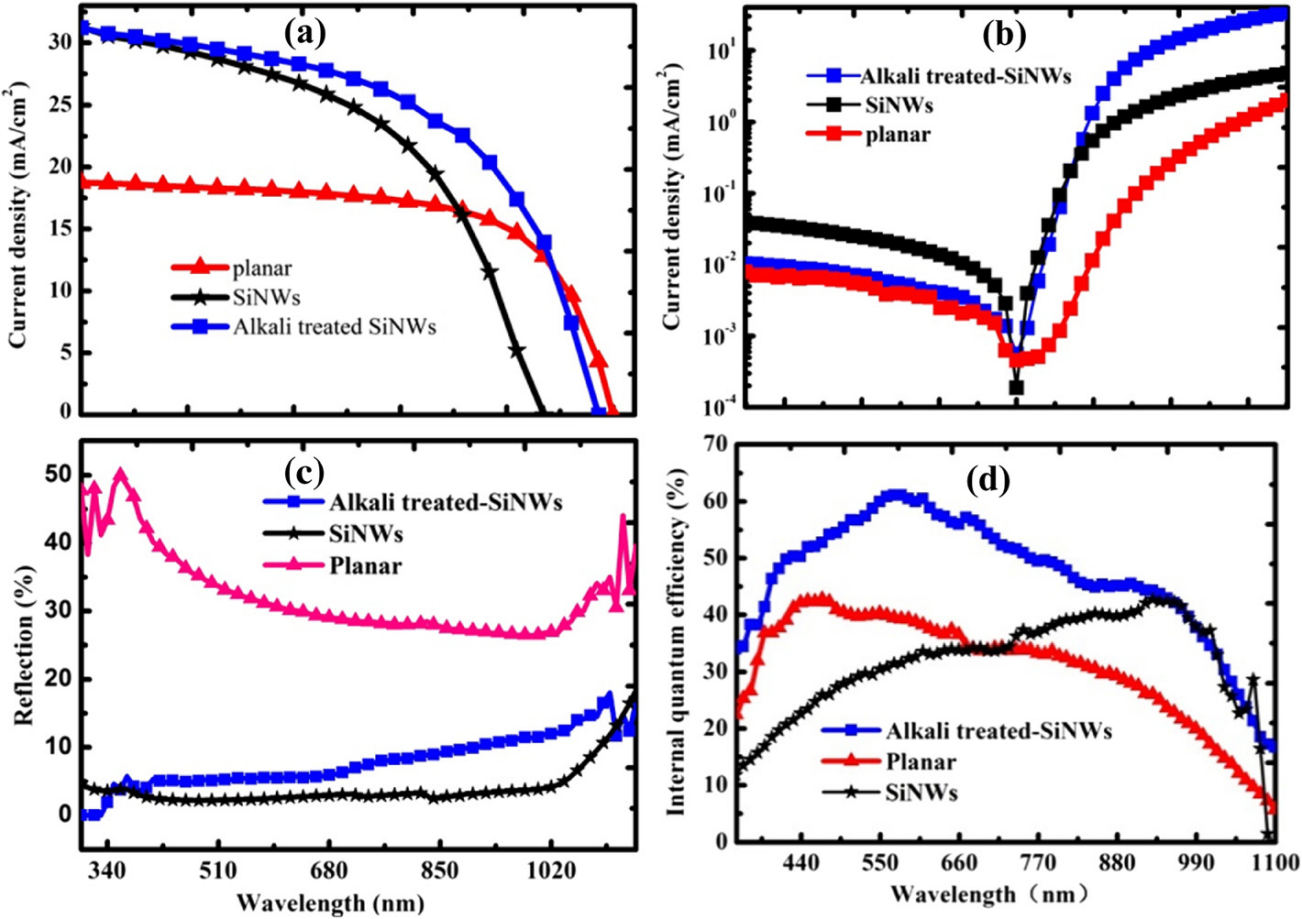
Characteristics of hybrid PEDOT:PSS/SiNWs solar cells. **a** Current density-voltage (*J*-*V*) curves of three devices: planar Si/PEDOT:PSS, SiNWs/PEDOT:PSS, and alkali treated-SiNWs/PEDOT:PSS. **b** Dark current density-voltage (*J*-*V*) curves in **a. c** Optical reflection spectra of various solar cells: SiNWs, alkali-treated SiNWs with alkali treatment and planar device. **d** IQE spectra of the three devices in **a**

Figure 1a shows the current density-voltage (*J*-*V*) curves of the three devices: Si planar/PEDOT:PSS, PEDOT:PSS/SiNWs, and alkali treated-PEDOT:PSS/SiNWs. The electrical output characteristics are summarized in Table 1. The *J*_sc_ of the alkali-treated SiNW device is increased from 29.8 to 34.0 mA/cm^2^, reflecting a 14.1 % enhancement compared to SiNW solar cell, and the open-circuit voltage (*V*_oc_) is increased from 0.44 to 0.49 V, reflecting a 11.4 % enhancement compared to SiNW solar cell. With the improvements of *J*_sc_, *V*_oc_, and FF (from 51 to 59 %), the conversion efficiency (PCE) of alkali-treated SiNW solar cell is increased from 6.0 to 9.1 %, reflecting a 51 % enhancement compared to SiNW ones. Measurements were repeated with different sets of samples, and highest *V*_oc_ and *J*_sc_ had been consistently obtained with the alkali-treated SiNWs solar cell. In general, the overall performance of alkali-treated SiNWs solar cells is improved and the alkali treatment processing plays a critical role on the device performance.

**Table 1:** Photovoltaic properties of the hybrid Si planar, SiNWs, and alkali-treated SiNWs solar cells

Sample	*V*_oc_ (V)	*J*_sc_ (mA/cm^2^)	FF (%)	PCE (%)
SiNWs	0.44	29.8	51	7.3
Alkali-treated SiNWs	0.49	34.0	59	9.1
Planar	0.50	17.1	65	6.0

To further investigate the reasons for the improved efficiency, it is important to gain insight into the correlation between *J*_sc_ enhancement and carrier collection enhancement in the active layer. The IQE is measured as shown in Fig. 1d. One can see that IQE of the alkali-treated SiNW device is above 50 % over a wide wavelength range between 400 and 1000 nm, but SiNWs and planar devices have a maximum IQE of 40 % at 400 and 550 nm, severely decreases from 400 to 1100 nm. Compared with PEDOT:PSS/SiNW solar cells and PEDOT:PSS/Si planar solar cells, IQE of alkali-treated SiNWs device is the highest and reaches a peak value of 60 % at the wavelength of 600 nm. The IQE of planar solar cells is the lowest and reaches its peak value of 37 % at the wavelength of 500 nm. In short, the photoresponse for the alkali-treated SiNWs device is significantly better than that of SiNWs and planar one.

To further understand the limiting factors of the device performance, *J*-*V* characteristics of the devices in the dark are measured as shown in Fig. 1b. By comparing the dark-current measurement results, one can find that the SiNW/PEDOT:PSS with alkali treatment possesses small reversed saturated current compared to that of one without alkali treatment, which suggests that the recombination of carriers is reduced [22]. In our work, the devices possess the same materials and the analogue device structure; the difference should be caused by the alkali treatment. The results indicate that introduction of alkali treatment processing could suppress the recombination of photogeneration carriers,

Figure 1c shows the reflectance spectra of planar, SiNWs, and alkali-treated SiNWs, respectively. The reflectance of alkali-treated SiNWs is slightly higher than the area densities of the NWs that are accordingly decreased. Therefore, alkali treatment could not contribute to the light absorption, so the additional enhanced *J*_sc_ of alkali-treated device must step from the enlarged junction area.

**Fig. 2:**
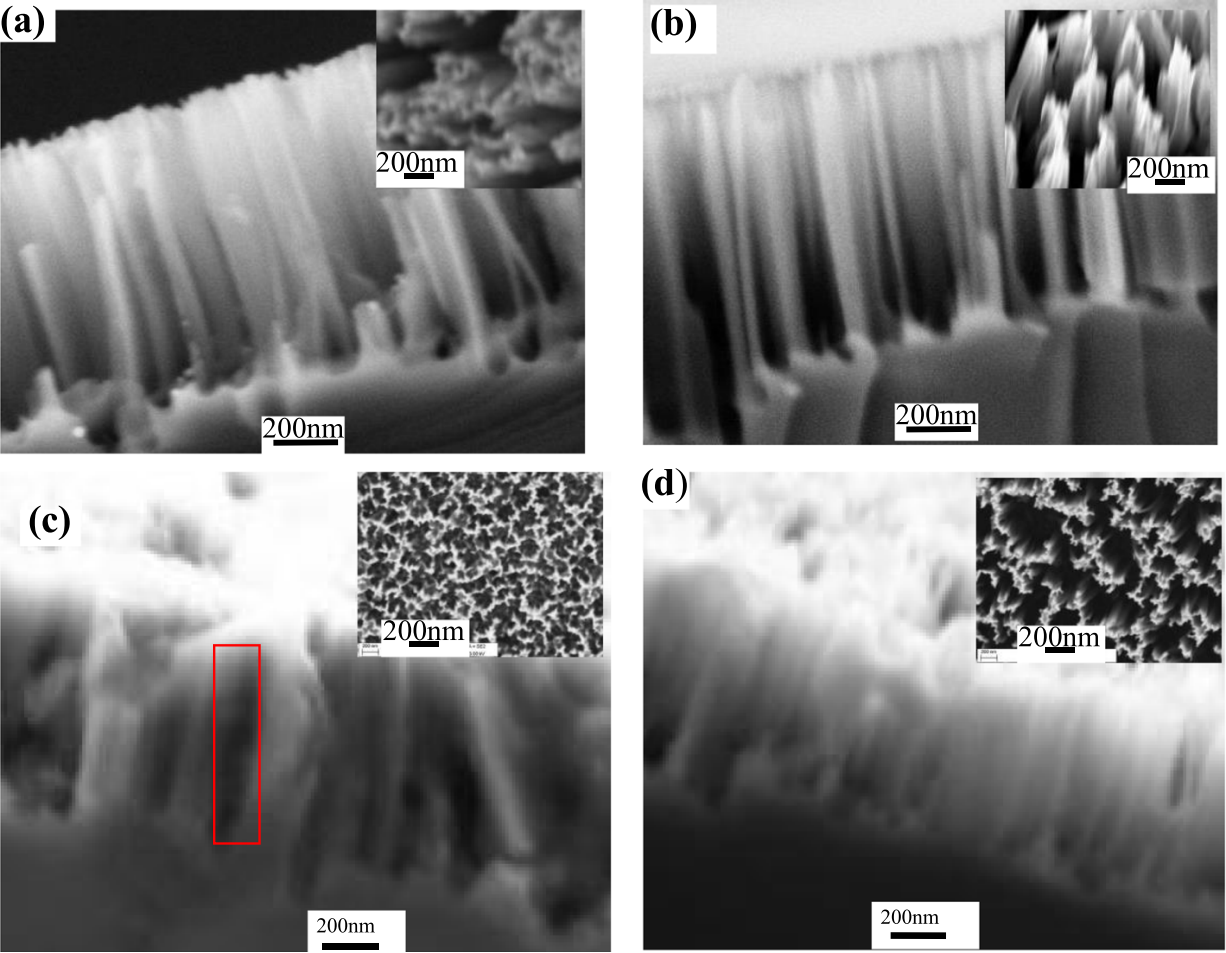
**a** Cross-sectional and top-view (tilted 15°) SEM image of SiNWs. **b** Cross-sectional and morphology SEM image of SiNWs with alkali treatment (tilted 15°). **c** Morphology and cross-sectional SEM image of PEDOT:PSS-coating SiNWs, the void with the *red mark*. **d** Morphology and cross-sectional SEM image of PEDOT:PSS-coating SiNWs with alkali treatment

Figure 2 includes the cross-sectional field emission SEM image and morphology of SiNWs and PEDOT:PSS-coating SiNWs. It can be seen from Fig. 2a that nanowires with a uniform height of about 1000 nm are vertically aligned on the Si substrate. The inset of Fig. 2a is the top-view SEM image of the original SiNWs. In addition, Fig. 2b also shows the cross-sectional SEM image and morphology of the alkali-treated SiNWs (inset). Therefore, the alkali treatment processing clearly changes the morphology of SiNWs and can enable to sparse and taper SiNWs. To investigate the effect of alkali treatment on the infiltration of polymer into the space between the nanostructures, the morphology of PEDOT:PSS-coated SiNWs is shown in Fig. 2c, d. Moreover, we can also see from Fig. 2c that the coated polymer is not completely infiltrated into the space between SiNWs. The incomplete infiltration leads to large hollow spaces between SiNWs which have not been covered with PEDOT:PSS (shown in the inset of Fig. 2c). For comparison, the coated polymers form the conformal coating of PEDOT:PSS over alkali-treated SiNWs as shown in Fig. 2d. It is possible to suggest in this scenario that alkali treatment plays an important role—forming the good core-shell heterojunctions and enhancing the junction area.

**Fig. 3:**
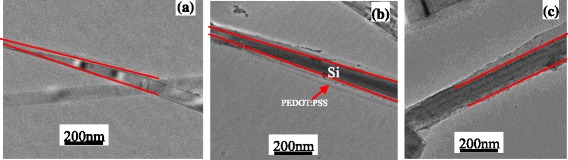
**a** TEM images of single SiNW with alkali treatment. **b** Single PEDOT:PSS coating SiNW with alkali treatment. **c** Single PEDOT:PSS coating SiNW without alkali treatment

Figure 3a shows the TEM image of single SiNW with alkali treatment. It can be seen from Fig. 3a that the mono-crystal SiNW can be tapered by alkali treatment. One can see from Fig. 3b that PEDOT:PSS film wraps the wire completely, and the film thickness is about 40~50 nm. For comparison, the TEM image of the PEDOT:PSS-coating SiNWs without alkali treatment is shown in Fig. 3c; it can be seen that the PEDOT:PSS film unevenly wraps the wire incompletely and inhomogenously. Therefore, alkali treatment processing plays an important role in the infiltration of polymer into SiNWs and the core/shell formation of SiNW/PEDOT:PSS.

**Fig. 4:**
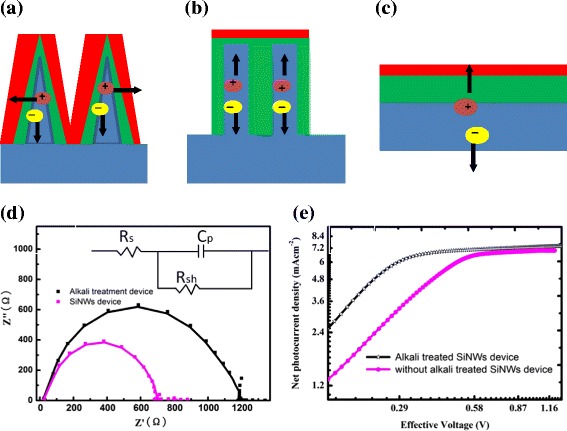
Schematic of photogenerated carriers transporting for **a** alkali-treated SiNWs device, **b** SiNWs device, and **c** Si planar device. **d** Nyquist plots of PEDOT:PSS/SiNWs and alkali treated-PEDOT:PSS/SiNWs solar cells at applied bias (0.4 V) under dark conditions; the *inset* is the equivalent circuit employed to fit the IS data. In all plots: *solid lines* fit to the model in the *inset* and the *dashed line* actual data points. **e** Photocurrent density versus effective voltage (*J*_ph_-*V*_eff_) characteristics of devices with alkali-treated and without alkali-treated devices

The core-shell heterojunctions change the direction of charge transporting shown in Fig. 4a. Compared to the planar (Fig. 4c) and SiNW (Fig. 4b) device, alkali-treated devices with good core-shell heterojunction configuration have large effective junction area. The photogenerated electron-hole pairs can be fast separated in the radial direction, thus minimizing the impact of carrier recombination and shortening their transport paths to collect them losslessly. The core-shell nanoheterojunctions could promote fast separating and exacting of the photogenerated carriers [23].

For the planar and SiNWs device, the distance of photogenerated carriers in the silicon substrate transporting to the top contact is about several micrometers, which is the length of SiNWs. The poor carrier collection efficiency in the deep region of the silicon substrate causes low photocurrent and low IQE [24].

To reveal how the EHP can be fast separated and collected in the core-shell heterojunctions, and to further understand the related mechanism for the enhanced performance of alkali treated devices, we investigated the charge dissociation probabilities in the alkali treatment devices, which indicates the charge collection efficiency and the charge recombination reducing under working condition [25]. Figure 4e shows the *J*-*V* characteristics in a wide reverse bias range under AM 1.5G illumination. The results are plotted as the net photocurrent (*J*_ph_ = *J*_L_−*J*_D_) dependence on the effective applied voltage *V*_eff_, where *J*_L_ and *J*_D_ are the current density under illumination and in the dark, respectively, *V* is the applied voltage, and *V*_o_ is the compensation voltage at which *J*_ph_ = 0.

At a large reverse voltage (*V*_eff_ = 1.2 V), *J*_ph_ reaches saturation for both devices. Noticeably, with increasing effective voltage, the saturation photocurrent (*J*_ph_) in the device with alkali treatment is reached earlier than that in the device without alkali treatment, suggesting that the cell with alkali treatment has the better charge transportability. These results suggest that the charge collection efficiency of the alkali-treated device was markedly enhanced, compared with the device without alkali treatment.

To have a deeper insight into the mechanism of the alkali treatment on the SiNWs cell performance, impedance spectroscopy (IS) has been systematically analyzed. IS is an effective method to analyze the solar cells, in particular, using it to analyze the recombination of carriers [26, 27]. From IS, we can extract the series resistance (*R*_s_) and the charge recombination resistance (*R*_rec_) in the cell. Nyquist plots of the cells with alkali treatment and without alkali treatment cells at 0.4 V are shown in Fig. 4e. The IS are dominated by a large semicircle. In this case, the inset of Fig. 4d is an equivalent circuit with one RC element which is employed to fit the IS data. We have conducted performance fitting and simulation of this data for an equivalent circuit shown in the inset. The observed parameters of *R*_s_, *R*_sh_, and *C*_p_ values are presented in Table 2. The analysis indicates that the *R*_s_ of the cells does not obviously change with alkali treatment; contact resistance *R*_s_ of SiNWs solar cell is about 12.67 Ω, similar to that (14.39 Ω) of the device with alkali treatment. Interestingly, the *R*_rec_ of the cell is significantly increased (from 732 to 1251 Ω) at applied bias when alkali treatment processing is introduced. It is well known that the *R*_rec_ explicitly relates to the electron and hole recombination within the solar cells under forward bias, which is associated with the interface charge transport process. The enhanced *R*_rec_ suggests that the interface recombination is reduced. Meanwhile, the reduced recombination is beneficial to improve *V*_oc_ which agrees with the variation tendency of the *V*_oc_ in Fig. 1a. On the other hand, the value of *C*_p_ in the device with alkali treatment is 5.6 E−5 F/cm^2^, much larger than that in the device without alkali treatment (4.2 E−5 F/cm^2^). The value of *C*_p_ is related to an ideal capacitor and suggests electron density of states [28]. The *C*_p_ enhancement shows that the interface capacitance between alkali-treated SiNWs and PEDOT:PSS is more perfect electrically than that of the device without alkali treatment; this is consistent to the reduced *J*_0_. The higher capacitance value may be attributed to the well-organized PEDOT:PSS/SiNW morphology facilitating the higher charge storage per unit increment of voltage. The origin of this variation can be interface states which are discharged under bias voltage. It is possible to suggest in this scenario that alkali treatment plays an important role—the shell layer of PEDOT:PSS could passivate the dangling bonds of SiNWs surface and thus reducing the effects of charges recombination by suppressing the effects of interfaces states, which results in the higher charge storage per unit increment of voltage [29]. This is consistent to the reduced of *R*_rec_. The mechanism of efficiency enhancement by terminating the dangling bonds to reduce the interfaces states has been proved by Bashouti et al. [30]. Further investigation will be done in our future work.

**Table 2:** Parameters of SiNWs and alkali-treated SiNWs solar cells determined by IS measurements under dark conditions

	Rs/Ω	Rsh/KΩ	Cp (10^−5^F)
SiNWs	14.67	712	0.86
Alkali-treated SiNWs	12.39	1231	8.94

**Fig. 5:**
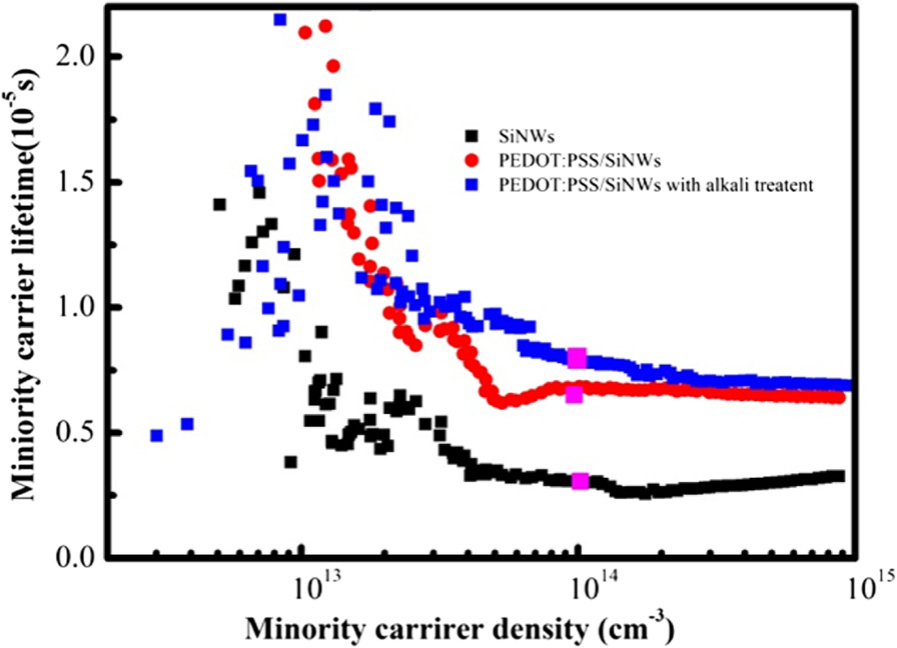
Effective minority carrier lifetimes at an injection level of 10^14^ cm^−3^

In order to understand the passivating performance of PEDOT:PSS, the effective minority carrier lifetime (*τ*_eff_) measurements of PEDODT:PSS/SiNWs and PEDODT:PSS/SiNWs with alkali treatment were performed (shown in Fig. 5) by the use of the quasi-steady state photoconductance (QSSPC) method using a Sinton Instruments WCT-120 apparatus [31]. *τ*_eff_ could give direct information on the surface passivation quality and is used to characterize the influence of the surface which represents the combined impact of all of the competing recombination channels. This result confirmed our assumption that the increase of *τ*_eff_ is due to PEDOT:PSS passivation (from 2.82 to 6.76 μs). Meanwhile, our results show that introduction of alkali treatment process could further improve the lifetime of the device (from 6.76 to 7.94 μs) due to the reduction of the surface recombination.

## Conclusions

In summary, the SEM and TEM showed that alkali treatment is well-suited for the hybrid Si/polymer solar cell since anisotropic etching can taper and spare the SiNWs which allows for conformal polymer coating to form the good core-shell heterojunctions, which results in fast collecting and separating EHPs. This hybrid structure can reduce carrier recombination ratio due to the short transportation distance of carriers in NWs and passivation of PEDOT:PSS, leading to enhanced carrier collection efficiency at the electrodes. This report emphasizes the importance of the alkali treatment process application in Si hybrid solar cells.

## References

[1] He L, Jiang CY, Wang H, Lai D (2012). High efficiency planar Si/organic heterojunction hybrid solar cells. Appl Phys Lett.

[2] Masahiro O, Tang ZG, Ryo I, Gotou T, Keiji U, Shirai H (2012). Efficient crystalline Si/poly(ethylene dioxythiophene):poly(styrene sulfonate): graphene oxide composite heterojunction solar cells. Appl Phys Express.

[3] Liu QM, Masahiro O, Tang ZG, Ryo I, Keiji U, Shirai H (2012). Highly efficient crystalline silicon/Zonyl fluorosurfactant-treated organic heterojunction solar cells. Appl Phys Lett.

[4] Abdul MS, Muhammad NA, Han-don U, Sang-won J, Koun CH, Sang-woo K, Jung-ho L (2012). A stamped PEDOT:PSS–silicon nanowire hybrid solar cell. Nanotechnology.

[5] Shiu SC, Chao JJ, Hung SC, Yeh CL, Lin CF (2010). Morphology dependence of silicon nanowire/poly(3, 4-ethylenedioxythiophene):poly(styrenesulfonate) heterojunction solar cells. Chem Mater.

[6] Zhang FT, Song T, Sun BQ (2012). Conjugated polymer-silicon nanowire array hybrid Schottky diode for solar cell application. Nanotechnology.

[7] Lu WH, Chen Q, Wang B, Chen LW (2012). Structure dependence in hybrid Si nanowire/poly(3,4-ethylenedioxythiophene):poly(styrenesulfonate) solar cells: understanding photovoltaic conversion in nanowire radial junctions. Appl Phys Lett.

[8] Thiyagu S, Hsueh CC, Liu CT, Syu HJ, Lin TC, Lin CF (2014). Hybrid organic-inorganic heterojunction solar cells with 12 % efficiency by utilizing flexible film-silicon with a hierarchical surface. Nanoscale.

[9] Yu P, Tsai CY, Chang JK, Lai CC, Chen PH, Lai YC, Tsai PT, Li MC, Pan HT, Huang YY, Wu CI, Chueh YL, Chen SW, Du CH, Horng SF, Meng HF (2013). 13 % efficiency hybrid organic/silicon-nanowire heterojunction solar cell via interface engineering. ACS Nano.

[10] Pietsch M, Bashouti M, Christiansen S (2013). The role of hole transport in hybrid inorganic/organic silicon/poly(3,4-ethylenedioxy-thiophene):poly(styrenesulfonate) heterojunction solar cells. J Phys Chem C.

[11] Zhang J, Song T, Shen X, Yu X, Lee ST, Sun B (2014). A 12 %-efficient upgraded metallurgical grade silicon–organic heterojunction solar cell achieved by a self-purifying process. ACS Nano.

[12] He L, Jiang CY, Wang H, Lai D (2011). Simple approach of fabricating high efficiency Si nanowire/conductive polymer hybrid solar cells. IEEE Electron Device Lett.

[13] Liu Y, Zhang Z, Xia Z, Zhang J, Liang F, Li Y, Song T, Yu X, Lee ST, Sun B (2016). High performance nanostructured silicon-organic quasi p-n junction solar cells via low-temperature deposited hole and electron selective layer. ACS Nano.

[14] Zhang Y, Cui W, Zhu Y, Zu F, Liao L, Lee ST, Sun B (2015). High efficiency hybrid PEDOT:PSS/nanostructured silicon Schottky junction solar cells by doping-free rear contact. Energy Environ Sci.

[15] Jeong S, Garnett E, Wang S, Yu Z, Fan S, Brongersma M, Mcgehee M, Cui Y (2012). Hybrid silicon nanocone–polymer solar cells. Nano Lett.

[16] Bai F, Li M, Huang R, Li Y, Trevor M, Musselman K (2014). A one-step template-free approach to achieve tapered silicon nanowire arrays with controllable filling ratios for solar cell applications. RSC Adv.

[17] Jung Y, Guo Z, Jee SW, Um HD, Lin KT, Lee JH (2010). A strong antireflective solar cell prepared by tapering silicon nanowires. Opt Express.

[18] Gong X, Jiang Y, Li M, Liu H, Ma H (2015). Hybrid tapered silicon nanowire/PEDOT:PSS solar cells. RSC Adv.

[19] Subramani T, Syu H, Liu C, Hsueh C, Yang S, Lin C (2016). Low-pressure-assisted coating method to improve interface between PEDOT:PSS and silicon nanotips for high-efficiency organic/inorganic hybrid solar cells via solution process. ACS Appl Mater Interfaces.

[20] Peng K, Wang X, Wu XL, Lee ST (2009). Fabrication and photovoltaic property of ordered macroporous silicon. Appl Phys Lett.

[21] Peng K, Xu Y, Wu Y, Yan Y, Lee ST, Zhu J (2005). Aligned single-crystalline Si nanowire arrays for photovoltaic applications. Small.

[22] Wang X, Peng K, Pan X, Chen X, Yang Y, Li L, Meng XM, Zhang WJ, Lee ST (2011). High-performance silicon nanowire array photoelectrochemical solar cells through surface passivation and modification. Angew Chem Int Ed.

[23] Tsai SH, Chang HC, Wang HH, Chen SY, Lin CA, Chen SA, Chueh YL, He JH (2011). Significant efficiency enhancement of hybrid solar cells using core-shell nanowire geometry for energy harvesting. ACS Nano.

[24] Liang ZC, Zeng F, Song H, Shen H (2013). Effect of porous Si and an etch-back process on the performance of a selective emitter solar cell. Sol Energy Mater Sol Cells.

[25] He Z, Zhong C, Huan X, Wong WY, Wu H, Chen L, Su S, Cao Y (2011). Simultaneous enhancement of open-circuit voltage, short-circuit current density, and fill factor in polymer solar cells. Adv Mater.

[26] Braña AF, Forniés E, López N, García BJ (2015). High efficiency Si solar cells characterization using impedance spectroscopy analysis. J Phys Conf Ser.

[27] Yadav P, Tripathi B, Pandey K, Kumar M (2015). Investigating the charge transport kinetics in poly-crystalline silicon solar cells for low-concentration illumination by impedance spectroscopy. Sol Energy Mater Sol Cells.

[28] Garcia-Belmonte G, Boix PP, Bisquert J, Sessolo M, Bolink HJ (2010). Simultaneous determination of carrier lifetime and electron density-of-states in P3HT:PCBM organic solar cells under illumination by impedance spectroscopy. Sol Energy Mater Sol Cells.

[29] Chal P, Shit A, Nandi AK (2016). Dye-sensitized solar cell from a new organic n-type semiconductor/polyaniline composite: insight from impedance spectroscopy. J Mater Chem C.

[30] Bashouti YM, Pietsch M, Brönstrup G, Sivakov V, Ristein J, Christiansen S (2014). Heterojunction based hybrid silicon nanowire solar cell: surface termination, photoelectron and photoemission spectroscopy study. Prog Photovolt Res Appl.

[31] Sinton RA, Cuevas A (1996). Contactless determination of current-voltage characteristics and minority carrier lifetimes in semiconductors from quasi-steady-state photoconductance data. Appl Phys Lett.

